# Expertise of the Robert Koch Institute for the first prevention report: background, aims and approach

**DOI:** 10.17886/RKI-GBE-2018-053

**Published:** 2018-05-16

**Authors:** Anne Starker, Susanne Jordan, Claudia Diederichs, Antje Wienecke

**Affiliations:** Robert Koch Institute, Berlin, Department of Epidemiology and Health Monitoring

The Preventive Health Care Act established the National Prevention Conference (NPC), a working group of the umbrella organisations for health, accident, pension and long-term care insurance, as well as private health insurance and long-term care insurance. The NPC has been given the task of developing and maintaining a national prevention strategy. This includes agreeing on cross-provider federal framework recommendations on health promotion and disease prevention as well as the associated documentation and reporting obligations. These obligations anticipate the preparation of a report on developments in health promotion and disease prevention (prevention report) every four years. The first report is due to be published on 1 July 2019.

The prevention report intends to document, review the performance and evaluate health promotion and disease prevention measures over time. The German Federal Framework Recommendations stipulate that prevention reports should supply information on the services provided and experiences related to achieving common goals and cooperation. In addition, these reports should also include results from the health monitoring undertaken at the Robert Koch Institute (RKI). Therefore, the German Federal Ministry of Health has commissioned the RKI to compile an expertise that brings together various epidemiological data. The expertise is to describe the health situation of the population living in Germany considering socio-economic and gender-related influences and to derive population-wide and target group-specific prevention needs and potentials. In doing so, data on various risks and protective factors and diseases are taken into account.

The following approach was chosen for the preparation of the expertise: In a feasibility study, selection criteria are defined for the eleven target groups named in the Federal Framework Recommendations; this should enable to describe the prevalence of risk and protective factors and diseases that are relevant to the various target groups. This approach will also involve taking into account important aspects such as Germany’s national health targets and current social developments such as the unequal distribution of health burdens among the population. In a second step, data availability is examined. Priority is given to the data of RKI’s health monitoring, but research will also be undertaken into external data sources. Furthermore, the report will also identify data gaps that may exist. This step will be followed by data preparation and descriptive and statistical data analyses. The results and the indication of prevention needs will finally be summarised in the expertise which will be available by the end of 2018.

